# Supplementary figures and images for: Progressive Shifts in the Gut Microbiome Reflect Prediabetes and Diabetes Development in a Treatment-Naive Mexican Cohort
Source: Front Endocrinol (Lausanne). 2021 Jan 8;11:602326. doi: 10.3389/fendo.2020.602326 (PMC7821428; doi:10.3389/fendo.2020.602326)

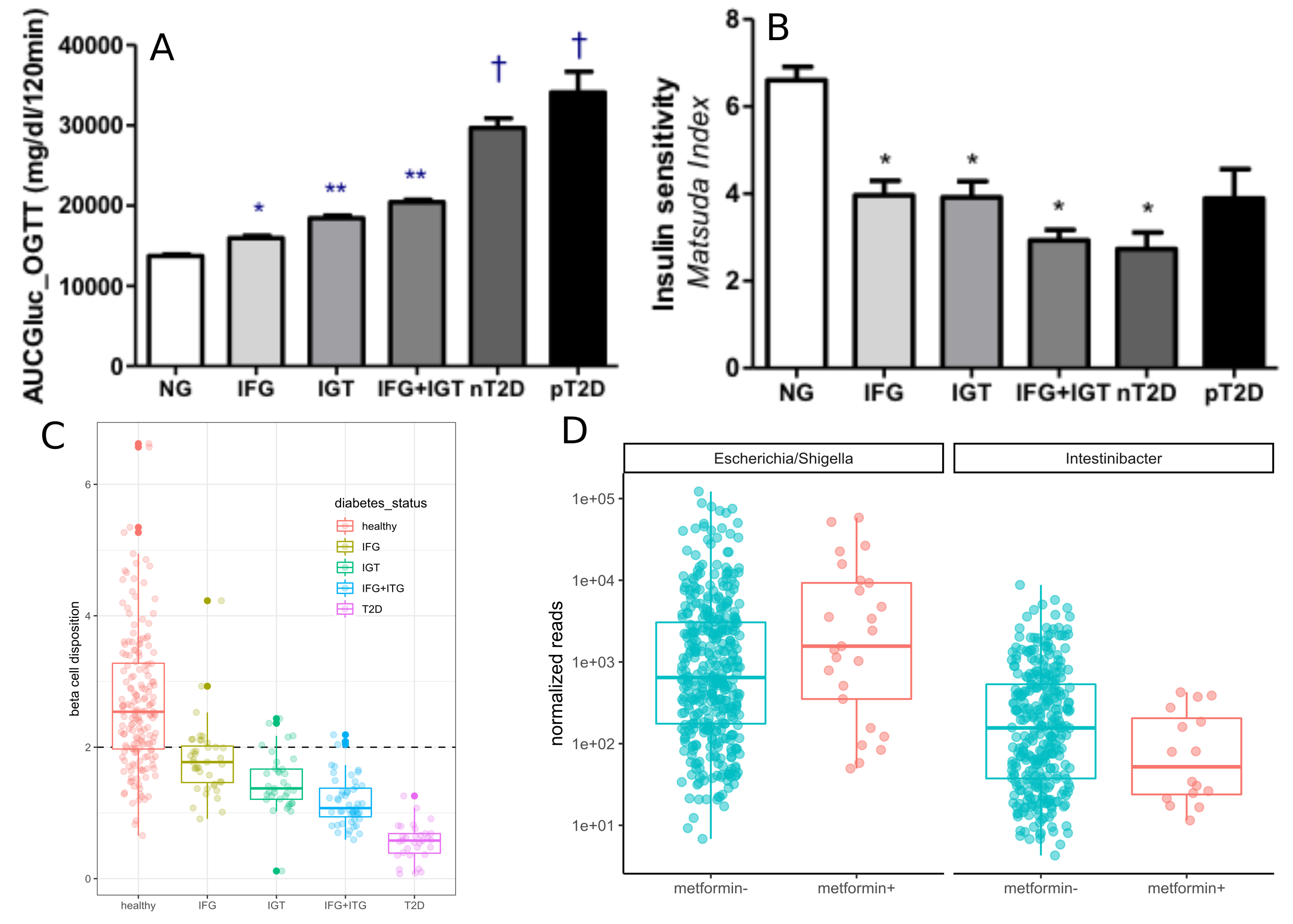

Supplement: Supplementary Figure 1 — (A) Increment of the area under the glucose curve stratified by metabolic group. “nT2D” denotes new (treatment-naive) T2D and pT2D denotes previous (treated) T2D. Bars denote the standard error of the mean. (B) Insulin sensitivity over the metabolic groups. Bars denote the standard error of the mean. (C) Beta cell disposition index stratified by metabolic group. The dashed line denotes the cutoff value 2 that was used to separate functional from non-functional beta cells. (D) Abundances of Escherichia and Intestinibacter stratified by metformin history. Superscripts denote the following comparisons in a t-test: *p < 0.01 vs. NG group, **p < 0.01 vs. NG and IFG groups, †p < 0.01 vs all groups. [file Image_1.tif]

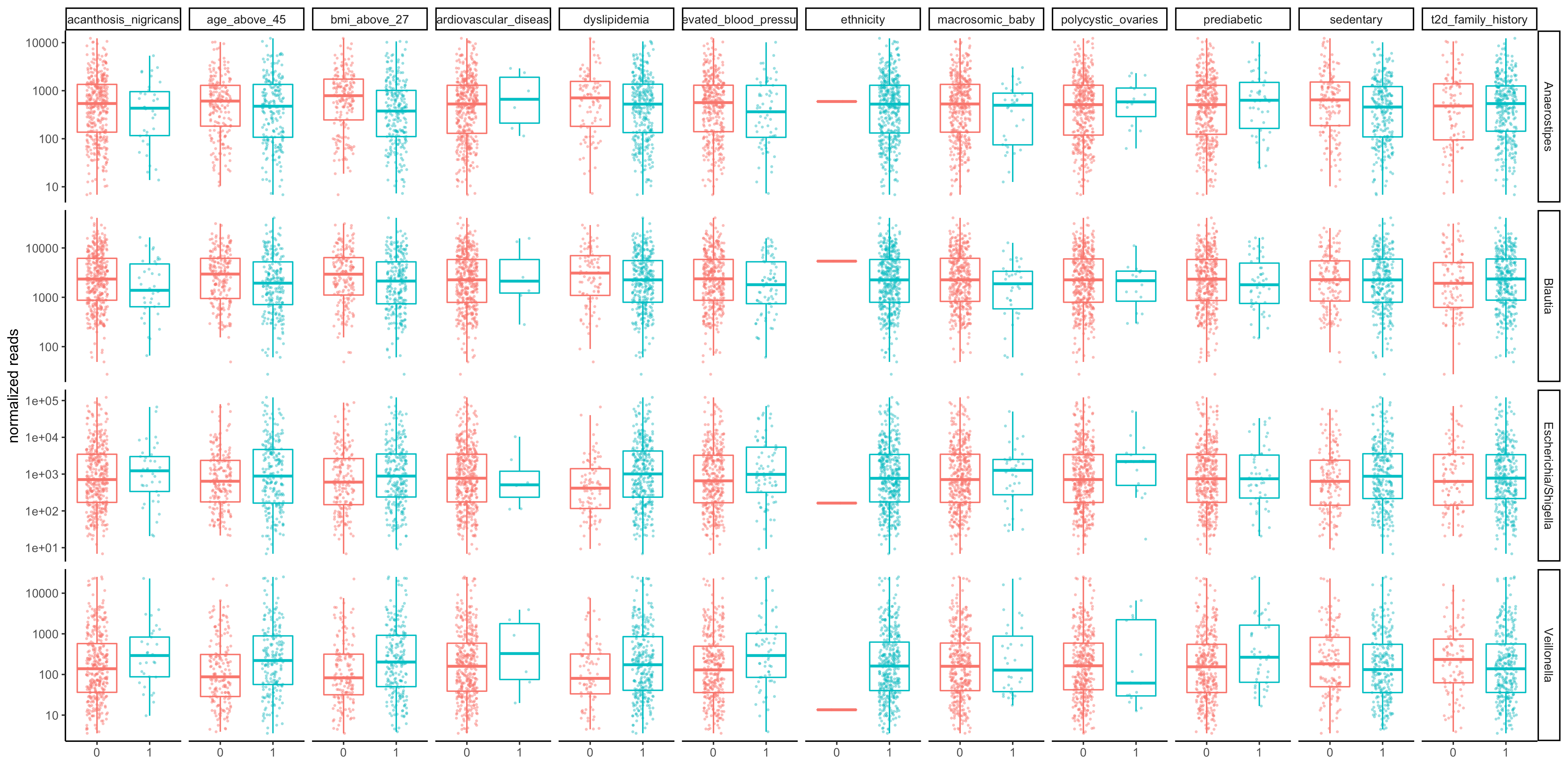

Supplement: Supplementary Figure 2 — Binary risk factors used to calculate the overall risk of developing T2D. Shown are bacterial abundances stratified by individual risk factors. Absence is denoted by zero and presence by 1. The following 3 risk factors were summarized into a single one (any of the 3 present) when calculating overall risk: polycystic ovary syndrome, cardiovascular disease, having a baby born with more than 4 kg of weight (macrosomia). Reads were normalized across samples as described in Methods. [file Image_2.tif]
